# Flavonoids-Macromolecules Interactions in Human Diseases with Focus on Alzheimer, Atherosclerosis and Cancer

**DOI:** 10.3390/antiox10030423

**Published:** 2021-03-10

**Authors:** Dana Atrahimovich, Dorit Avni, Soliman Khatib

**Affiliations:** 1 Lab of Natural Compounds and Analytical Chemistry, MIGAL–Galilee Research Institute, Kiryat Shmona 11016, Israel; Danaa@migal.org.il; 2 Department of biotechnology, Tel-Hai College, Upper Galilee 12210, Israel; 3 Lab of Sphingolipids, Bioactive Metabolites and Immune Modulation, MIGAL—Galilee Research Institute, Kiryat Shmona 11016, Israel; dorita@migal.org.il

**Keywords:** flavonoid, antioxidant, oxidative stress, inflammation, Alzheimer, atherosclerosis, cancer

## Abstract

Flavonoids, a class of polyphenols, consumed daily in our diet, are associated with a reduced risk for oxidative stress (OS)-related chronic diseases, such as cardiovascular disease, neurodegenerative diseases, cancer, and inflammation. The involvement of flavonoids with OS-related chronic diseases have been traditionally attributed to their antioxidant activity. However, evidence from recent studies indicate that flavonoids’ beneficial impact may be assigned to their interaction with cellular macromolecules, rather than exerting a direct antioxidant effect. This review provides an overview of the recent evolving research on interactions between the flavonoids and lipoproteins, proteins, chromatin, DNA, and cell-signaling molecules that are involved in the OS-related chronic diseases; it focuses on the mechanisms by which flavonoids attenuate the development of the aforementioned chronic diseases via direct and indirect effects on gene expression and cellular functions. The current review summarizes data from the literature and from our recent research and then compares specific flavonoids’ interactions with their targets, focusing on flavonoid structure–activity relationships. In addition, the various methods of evaluating flavonoid–protein and flavonoid–DNA interactions are presented. Our aim is to shed light on flavonoids action in the body, beyond their well-established, direct antioxidant activity, and to provide insights into the mechanisms by which these small molecules, consumed daily, influence cellular functions.

## 1. Introduction

**Figure 1:**
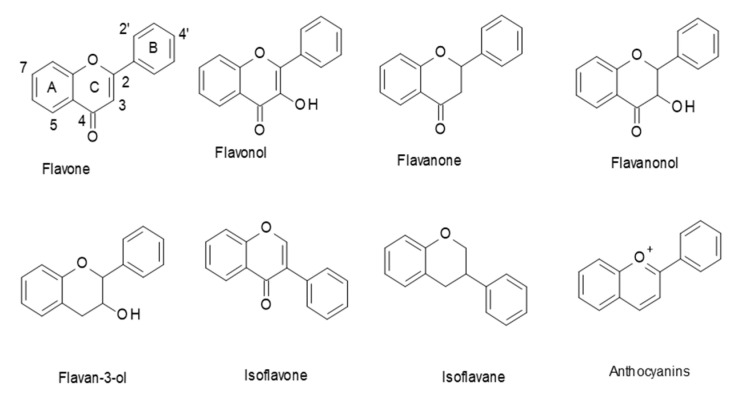
Structures of the main flavonoid subclasses.

Flavonoids are a class of polyphenols in plants that are widely consumed in our diet. They have a general C6–C3–C6 structural backbone, in which the two C6 units (Ring A and Ring B) are of a phenolic nature. Flavonoids can be divided into different subgroups, such as: flavones, flavonols, flavanones, flavanonols, flavan-3-ols, and anthocyanins (Figure 1). Whereas, in most flavonoids, ring B is attached at the C2 position of ring C, in some, such as isoflavones and isoflavans, ring B is connected at the C3 position [1].

Dietary flavonoids are natural products that are widely distributed in the plant kingdom. Many foods and beverages, such as fruits, vegetables, legumes, whole grains, chocolate, spices, tea, and wine, are rich sources of flavonoids [1]. Over decades, researchers and food manufacturers have become increasingly interested in flavonoids, due to their antioxidant properties, their great abundance in our diet, and their suggested role in the prevention of various diseases that are associated with OS, such as cancer, cardiovascular, and neurodegenerative diseases [2,3,4,5]. Recent literature provides growing evidence of flavonoids’ effects being mediated by mechanisms other than the classical antioxidant activity driven by their chemical property of donating an electron or chelating transition metals [6,7]. Exploring their fundamental modes of action could provide new insights into the mechanisms by which flavonoids influence biological functions.

## 2. Biological Activities of Flavonoids

### 2.1. Flavonoids as Antioxidants

With respect to their antioxidant activity, flavonoids are believed to prevent diseases that are related to OS via the direct scavenging of reactive oxygen species (ROS) through the donation of a hydrogen atom, activation of antioxidant enzymes, metal (such as iron and copper)-chelating activity, and alleviation of oxidative stress that is caused by nitric oxide (NO) [1,8,9,10,11]. Antioxidant activity, though, cannot be the sole explanation for the in vivo cellular effects of flavonoids’, since antioxidant activity is expressed at flavonoid concentrations that are above 10 μM, but their concentration in the circulation does not exceed 2 μM [12]. Dietary flavonoids are poorly absorbed from the intestine, highly metabolized, or rapidly eliminated. During the course of absorption, flavonoids are conjugated in the small intestine and later in the liver. This process mainly includes methylation, sulfation, and glucuronidation. This is a metabolic detoxification process that is common to many xenobiotics that restricts their potential toxic effects and facilitates their biliary and urinary elimination by increasing their hydrophilicity [13].

**Figure 2:**
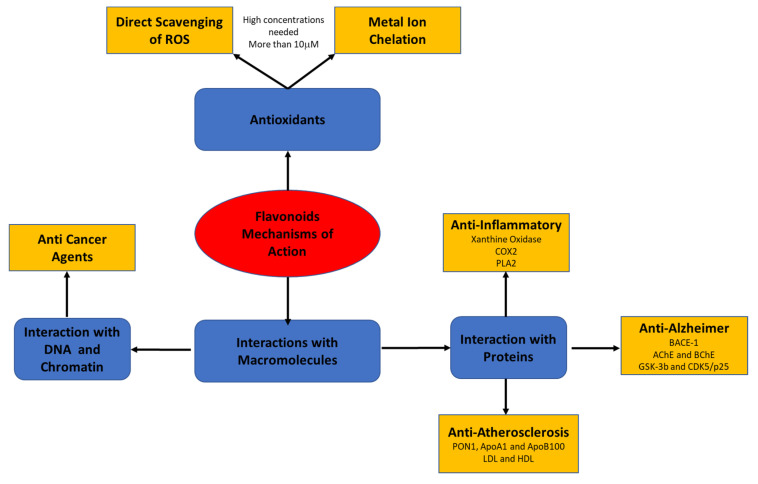
Flavonoids’ mode of action through their interaction with macromolecules.

Recent studies have suggested that the biological effects of flavonoids may be mediated by different mechanisms that have not yet been fully explored. The present review focuses on flavonoids’ mode of action through their interaction with macromolecules, such as lipoproteins, cell and serum proteins, and DNA and RNA (Figure 2).

### 2.2. Flavonoid Interactions with Macromolecules

#### 2.2.1. Flavonoid–Protein Interactions

Molecular interactions of proteins and nucleic acids with low-molecular-weight compounds are an area of fundamental interest [14]. At low concentrations, molecules, such as ions, metabolites, and osmolytes, may affect proteins, such as enzymes, receptors, antibodies, and transcription factors [15]. The effect may be at the structural, functional, or conformational levels [7]. Dietary flavonoids are a good example of small molecules that mediate cellular effects, which are central to intracellular signaling cascades [16]. The effects of flavonoid–enzyme complexes formed by flavonoids’ interaction with, for example, hydrolases, oxidases, and kinases, on the enzyme’s structure and activity have been widely explored. Investigations have suggested that flavonoids selectively interact with different components of protein kinases and alter their phosphorylation state, thus regulating multiple cell-signaling pathways [17]. Similarly, flavonoids have been found to act as ligands for nuclear receptors, causing their proliferation or activation and modulating energy homeostasis. Apigenin and kaempferol directly suppressed the interaction between estrogen-related receptor γ (ERRγ) and its coactivator peroxisome proliferators-activated receptor γ coactivator-1α (PGC-1α). In contrast, luteolin suppressed PGC-1α activity through promoting the degradation of PGC-1α, leading to suppressed ERRγ activity in HeLa cells [7,18]. Flavonoids, such as glabridin and glabrene, can also interact with, and modulate, the endogenous activities of estrogen receptors in human endothelial and smooth muscle cells, thereby may slow and even prevent cardiovascular diseases and the development of breast and ovarian cancers in post-menopausal women [19]. In addition, the flavonoids’ ability to interact with serum albumin and other serum proteins has also been investigated [20,21]. The reversible or irreversible protein–flavonoid interactions depend on pH, temperature, and the protein and flavonoid concentrations [22]. Although the biological fate of protein–flavonoid complexes in vivo is still unknown, flavonoids were found to affect various human diseases that were related to OS, such as cancer, and cardiovascular and neurodegenerative diseases [23,24,25].

##### Methods for Characterizing Flavonoid–Protein Interactions

Several studies have been performed to characterize the interactions between dietary flavonoids and proteins, mainly serum and food-related proteins, for example, serum albumins and β-casein [26,27,28,29,30]. Flavonoid–protein interactions mainly occur by noncovalent bonding that is derived from hydrophobic, van der Waals, hydrogen bridge-binding, and ionic interactions, which can change protein conformations and enzyme activities [31]. Noncovalent interactions between flavonoids and proteins are weak and reversible. Studies have also provided information on the covalent reactions between flavonoids and proteins. Flavonoids can easily oxidize and covalently react with the amino and thiol side chains of a protein by irreversible binding [32]. Numerous methods, mostly spectroscopic, have been developed to characterize the noncovalent interactions between flavonoids and proteins (Table 1) [33,34,35,36].

*UV-visible spectroscopy* is used to predict flavonoid–protein interactions and provide information on the nature of those interactions. Protein absorption at 280 nm is related to the aromatic amino acids tryptophan, tyrosine, and phenylalanine, which may be further stimulated upon interaction with flavonoids [37]. *Circular dichroism spectroscopy* is used for quantitative analysis of the conformational changes, α-helix and β-sheet changes, in proteins due to noncovalent interactions with small molecules, such as flavonoids [38]. *Fourier transform infrared spectroscopy* is also used to determine the changes in proteins’ secondary structure as a result of flavonoid interactions. This method allows for interpreting the secondary structure from the shape of the amide I band, located around 1650–1660 cm [38].

Thermodynamic properties of the binding interaction between flavonoids and proteins can be studied using *isothermal titration calorimetry*, a method that is based on measuring the heat that evolved during the molecular association [39]. Vichali et al. evaluated the binding interactions between four flavonoids (kaempferol, luteolin, quercetin, and resveratrol) and human serum albumin and glutathione S-transferase Pi isoform 1 using *Taylor dispersion surface plasmon resonance (SPR)*—a highly sensitive, label-free technique to study the noncovalent interactions of biomolecules, especially between proteins, and between proteins and small molecules [40].

*Tryptophan (Trp)-fluorescence quenching assay* is another sensitive, selective, and widely used method for determining interactions between flavonoids and proteins [21,41,42]. The excitation of proteins at 280–290 nm induces the emission of fluorescence in the range of 340–350 nm due to the presence of Trp. fluorescence quenching in this range can be attributed to flavonoid binding. While using this method, the quenching mechanism—static (complex formation between polyphenol and protein) or dynamic (collision of fluorophore with the quencher)—can be determined using the Stern–Volmer equation and calculating the Stern–Volmer constant and quenching rate constant. For static quenching, the binding constant and number of binding sites in the protein molecule can be calculated, and then thermodynamic properties can be characterized. Finally, *docking calculations* can be used to predict the fit of the evaluated ligand within the protein, where the shape is complementary to the binding site. Computational modeling complements the experimental data on flavonoid–protein binding and it allows for large-scale screening for different protein targets selected from the structures that are available in the Protein Data Bank (PDB) [43].

#### 2.2.2. Flavonoid Interactions with DNA and Chromatin

There is a great deal of evidence in the scientific literature of genome regulation by flavonoids via gene-expression and chromosomal alterations [24,51], although the precise mechanism of action remains unclear [48,52]. Flavonoids, such as quercetin and EGCG, have been shown to penetrate cell membranes and accumulate in the nucleus of human intestinal and hepatic cells [53,54]. The structure of quercetin allows for hydrophobic-nature-type intercalation of its most hydrophobic segment into the interior of the DNA helix [55]. Quercetin intercalates with DNA and RNA duplexes and preferentially binds to triplex and tetraplex DNA in human prostate cancer cells (DU 145) [53]. Although the same number of OH groups, which are mainly involved in the hydrogen-transfer mechanism, are present in kaempferol and luteolin, the latter exhibits slightly higher affinity to DNA. This might be due to the presence of OH at its 3′ position. Structure–activity relationships in flavonoid–DNA interactions have indeed been widely detected. It is proposed that flavonoids’ affinity for DNA increases along the same sequence as that exhibited by their biological activity [44]. Upon DNA treatment with EGCG or quercetin, various effects, including DNA damage, in human peripheral lymphocytes, were noted [56,57]. Studies show that EGCG inhibits the activities of various chromatin proteins, such as cAMP-response element-binding protein, DNA polymerase, DNA methyltransferase, and DNA topoisomerase in human lungs and colorectal adenoma cells and in mice liver, lungs, and kidney [6,24]. These reactions are likely affected by EGCG binding to the DNA and RNA, or to the proteins that are attached to nucleic acids in various types of interaction.

While the interactions of flavonoids, such as resveratrol, quercetin, EGCG, and genistein, with DNA are known, the precise location of the flavonoid-binding sites on the DNA, the mode of interaction, and its function in the genome are not fully understood.

##### Methods for Characterizing Flavonoid–DNA Interactions

Covalent binding of small molecules to DNA was first observed in the early 1980s [58]. After the covalent binding of [^14^C]quercetin to DNA was determined, it was argued that flavonoids have conflicting biochemical activities (mutagenic effect on the one hand, and anticarcinogenic effect on the other) [44]. In addition to covalent binding, flavonoids can interact with DNA by intercalation, groove-binding, and backbone-binding. Several methods have been used to elucidate the noncovalent interactions between flavonoids and DNA, including electrochemical and SPR techniques, linear dichroism, absorption, fluorescence, and nuclear magnetic resonance spectroscopies [44,45,46]. The binding of 10 aglycones and flavonoid glycosides with DNA duplexes was investigated while using electrospray ionization mass spectrometry (ESI-MS) [47]. ESI-MS analysis and SPR showed that exactly three molecules of EGCG bind to poly(dT) 18 mer single-stranded DNA oligomers via one hydroxyl group of the trihydroxyphenyl group in EGCG. Upon binding, the EGCG protected double-stranded DNA oligomers from melting to single-stranded DNA [59]. 

Today, computational simulation and spectroscopy are mainly adopted to explore biophysical information (e.g., interaction mode) on the interactions between flavonoids and DNA [60]. Experiments that were performed in recent years have suggested specific consensus DNA-binding sites for flavonoids. Quercetin, for example, binds to the dodecamer duplex sequence CGCGAATTCGCG, the unbound structure of which was solved many years ago (PDB ID: 1BNA) [61]. Currently, organism’s complete genome can be revealed using next-generation sequencing (NGS) technologies, such as Illumina or Sanger massively parallel sequencing machines. Moreover, following specialized protocols, it is possible to extract the DNA in specific regions or with specific functions and then use NGS to obtain the DNA sequence. Chem-seq (chemical affinity capture coupled with massively parallel DNA sequencing) is a new NGS application, which recently used to extract and sequence DNA regions that were bound to small molecules. This method allows for capturing chromatin regions bound to small molecules with no prior information, i.e., with an unbiased, nonspecific marker [49]. The latest studies have already illustrated the ability to isolate known drug–chromatin interactions using Chem-seq [49,50]. Atrahimovich et al. used the Chem-seq technique to characterize the interactions between quercetin and cellular DNA and demonstrated its subsequent effect on downstream transcription [48]. The results show that quercetin binds to monocytes’ chromatin and modulates the expression of genes that are involved in the cell cycle and cell development [48]. Using Chem-seq application, flavonoids interactions with DNA and chromatin may be determined to study its significance. This ability could be extremely important to medicine and human health, and beneficial to the design of appropriate dietary interventions and drugs for cancer treatment.

## 3. Flavonoids Attenuate Human Diseases via Direct Interactions with Proteins, Lipoproteins and DNA

### 3.1. Flavonoids Interactions with Key Proteins Involved in Inflammation

Inflammation characterizes the protective response of the immune system, involving the production of various proinflammatory cytokines and chemokines, which enhance the production of interferon-γ, proteases, NO, and ROS [62]. Cytokines also induce the expression of cyclooxygenase-2 (COX-2), an enzyme that catalyzes the production of prostaglandins (PGs), which are key mediators of inflammation [63]. Xanthine oxidase (XO) is another critical source of ROS that contributes to inflammation. Inflammatory conditions lead to increased XO levels and, thus, to increased ROS generation and peroxynitrite formation. Peroxynitrite is a powerful reactive nitrogen species (RNS) accompanied by OS, which is produced by the reaction of NO and superoxide radical [64].

Several mechanisms of action have been proposed to explain flavonoids’ anti-inflammatory activity in vivo, such as antioxidant activity and the modulation of the production of proinflammatory cytokines and gene expression [11]. Interestingly, flavonoids affect the inflammatory process not only by reducing the expression of cytokines and other related inflammatory markers, but also by interacting with proteins that are related to inflammation. Flavonoids have been shown to modulate the activity of arachidonic acid (AA)-metabolizing enzymes, such as phospholipase A2 (PLA2), COX, and lipoxygenase (LOX), and the NO-producing enzyme nitric oxide synthase (NOS). The inhibition of these enzymes by flavonoids reduces the production of AA, PG, leukotriene, and NO, which are crucial mediators of inflammation. Thus, flavonoid inhibition of these enzymes is definitely one of the important cellular mechanisms of anti-inflammation [65].

Quercetin was the first discovered flavonoid inhibitor of PLA2, from human neutrophils. Quercetin was shown to selectively inhibit group II secretory PLA2 [66]. Likewise, rutin selectively inhibited human PLA2-II from synovial fluid, while it was a weak inhibitor of human PLA2-I from pancreatic juice. When different flavonoids were compared for their ability to inhibit PLA2, small changes in the structure appeared to influence both overall PLA2 inhibition and group II selectivity. The position of the hydroxyl groups was found to be one important aspect of the C-ring-2, 3-double bond. The hydroxyl groups in the 3’ and 4’ positions on the B-ring seemed to be important for the selective inhibition of PLA2-II, whereas the 5-hydroxyl group on the A-ring, the unsaturation, and the 4-oxy on the C-ring seemed to be important for the overall ability of flavonoids to inhibit PLA2 activity [67]; the inhibition of PLA2 was very much dependent on hydroxyl groups position on rings A, B, and C, while hydroxyl groups in positions 5, 6, and 7 on the A-ring were assumed to be necessary for binding to PLA2s. Thus, quercetin, kaempferol, and galangin showed high inhibitory activity on PLA2, while naringin demonstrated a lower inhibitory activity [68].

COX produces PGs and thromboxanes and exists in at least two different isoforms, COX-1 and COX-2. COX-1 is a constitutive enzyme that is present in almost every cell type. While, COX-2 is an inducible enzyme that is highly expressed in the inflammation-related cell types, including macrophages and mast cells [69]. Because it produces PGs, COX-2 is closely associated with acute as well as chronic types of inflammatory disorders. Some flavonoids, such as luteolin, 3’,4’-dihyroxyflavone, galangin and morin, catechin, and epi-catechin, have been found to inhibit the rat renal medulla COX with IC50 of 100–130 μM [70]. In human thrombin-aggregated platelets, certain flavonoids, such as chrysin and apigenin, were revealed to be COX inhibitors with IC50 of 13 and 18 μM, while myricetin and quercetin at 10 μM exerts a strong inhibition of LOX. In particular, the reduction of the C-2, 3-double bond and glycosylation reduced the flavonoids inhibitory activities [71]. In-silico analysis demonstrated that quercetin could partially inhibit the COX-2 enzyme by binding to subunit A, which has peroxidase activity and serves as a ROS source [72].

D’mello et al. modeled the COX-2-inhibitory activity of some flavonoids [73], who used docking studies to determine which flavonoids act as COX-2 inhibitors. It was found that flavonoids have different types of binding patterns, which are similar to synthetic nonsteroidal anti-inflammatory drugs (NSAIDs). Myricetin and luteolin had catechol moiety with 3′, 4′-dihydroxy groups on the B-ring, and baicalein had the catechol-moiety on the A-ring, both being oriented toward the hydrophobic pocket of the enzyme and formed H-bonds with Tyr20, Tyr385, and Ser530. However, myricetin cannot act as a selective COX-2 inhibitor because it was inaccessible toward Arg513, an important residue for the selective inhibition of COX2. The importance of the catechol moiety was further confirmed by its similarity to the existing COX inhibitors [73]. Moreover, the effect of flavonoids on iNOS has been intensively studied. Several flavonoids, including apigenin, luteolin, and quercetin, were found to inhibit NO production in the macrophage cell line (RAW 264.7) that was treated with LPS/cytokine. However, they reduced NO production via downregulation of iNOS induction and not via the inhibition of iNOS by direct interaction [74]. The only exception was echinoisosophoranone, which significantly inhibited iNOS at sufficient concentrations [75]. While they may inhibit endothelial NOS or neuronal NOS, flavonoids are not efficient iNOS inhibitors. Interestingly, flavonoids have been found to be promising potent inhibitors of XO activity in humans. For example, quercetin and its glycosides displayed significant inhibitory activity against XO, thereby affecting ROS formation and the inflammation that is promoted by OS [76,77].

Generally, flavonoids may be mainly involved in the inflammation process via inhibition and regulation of enzymes that modulate pro-inflammatory cytokines or small molecules, such as ROS and RNS.

### 3.2. Flavonoids Interactions with Key Proteins in Alzheimer’s Disease (AD) 

AD is a widespread neurodegenerative disease, which is characterized by neurofibrillary tangles, senile plaques, and synaptic loss, eventually resulting in neuronal death [78,79]. AD is a form of dementia, which is characterized by progressive memory loss, a decline in language skills, and other cognitive impairments, and it most commonly affects the elderly [80]. AD’s etiology is unclear; however, a variety of factors are considered in the pathophysiology of the disease, such as the formation of amyloid β-protein (Aβ) plaques, low levels of acetylcholine, oxidative stress, and abnormal posttranslational modifications of tau protein [81,82]. The sequential cleavage of amyloid precursor protein forms aggregates of Aβ peptides of 39–43 amino acids, which stick to the neurons as insoluble amyloid plaques. Aβ is generated from the amyloid precursor protein by β-site amyloid precursor protein cleaving enzyme-1 (BACE-1, β-secretase) and γ-secretases [83,84]. Thus, the inhibition of BACE-1 is assumed to play an important role in the prevention of AD [85].

The neurotransmitter acetylcholine plays an important role in the process of learning and memory in the hippocampus. Two enzymes, acetylcholinesterase (AChE) and butyrylcholinesterase (BChE), are involved in the hydrolysis of acetylcholine, lowering its level during the development of AD. Therefore, the inhibition of AChE and BChE is a highly desirable strategy for the treatment of AD [86,87,88]. The clinically approved drugs tacrine, donepezil, galantamine, and rivastigmine improved short-term memory and cognitive levels via inhibition of AChE. The disadvantages of these drugs and their gradual side effects, such as peripheral side effects, hepatotoxicity, and gastrointestinal tract disorders, have encouraged researchers to develop more effective AChE inhibitors [89,90,91].

Flavonoids are promising natural products with neuroprotective potential, which either prevent the onset or slow the progression of age-related neurodegenerative diseases. The mechanism by which flavonoids prevent or slow the progression of AD might be via direct interaction with key enzymes that are involved in this disease [81,85,92,93,94,95]. Shimmyo et al. examined the potential of flavonols and flavones to inhibit BACE-1. They found that four flavonols: myricetin, quercetin, kaempherol, and morin, and one flavone: apigenin, directly inhibit BACE-1 enzyme activity in a concentration-dependent manner, with IC_50_ values of 2.8, 5.4, 14.7, 21.7, and 38.5 μM, respectively [95]. Studies in aged TASTPM transgenic mice (a model of AD) showed that the oral administration of (-)-epicatechin reduces Aβ pathology through indirect, noncatalytic BACE-1 inhibition and not through modulation of either α- or γ-secretase activity [96]. Epigallocatechin-3-gallate (EGCG) and curcumin were found to reduce Aβ-mediated BACE-1 upregulation in neuronal cultures, which, interestingly, increased the nonamyloidogenic processing of the amyloid precursor protein by enhancing α-secretase cleavage [95]. Pueyo et al. reviewed the literature on natural and synthetic flavonoids with AChE-inhibitory activity. They found 128 such flavonoids: 41 flavones, 21 flavanones, 35 flavonols, 25 isoflavones, and six chalcones. Among them, eight synthetic flavonoids inhibited AChE with IC_50_ < 100 nM. Three natural flavonoids, acaciin from *Chrysanthemum indicum* flowers, and desmethylanhydroicaritin and kaempferol from *Sophora flavescens* roots, inhibited AChE, with IC_50_ values of 3.2, 6.7, and 3.3 nM, respectively [97]. Orhan et al. screened various flavonoid derivatives for their inhibition of AChE and BChE. At a concentration of 1 mg/mL, quercetin was the most effective toward AChE, with 76.2% inhibition, and genistein showed the highest inhibition (65.7%) of BChE, followed by luteolin-7-*O*-rutinoside and silibinin (54.9% and 51.4%, respectively) [98,99]. In another study, *Citrus junos* had a significant inhibitory effect on AChE in vitro and in vivo, and the active compound was identified as naringenin, a major flavanone derivative [100]. Lee et al. examined the inhibitory effect of citrus flavanones on BACE-1, AChE, and BChE. Among all of the examined flavanones, hesperidin demonstrated the best inhibition of BACE-1, AChE, and BChE, with IC_50_ values of 10.02, 22.80, and 48.09 µM, respectively. Kinetic studies revealed that all the flavanones were noncompetitive inhibitors of BACE-1 and cholinesterase [101,102].

Hyperphosphorylation of tau proteins with subsequent accumulation as neurofibrillary tangles is a major contributor to cognitive dysfunctions and one of the earliest AD markers. Several kinases, such as GSK-3b and CDK5/p25, are known to contribute to the phosphorylation of tau proteins and they are implicated in the pathogenesis of AD. Flavonoids that inhibit the activities of several kinases can be used in AD prevention. Therapy with the flavonoid morin has been shown to reduce tau hyperphosphorylation in vitro and in vivo in the hippocampal neurons of transgenic animals (3xTg-AD mice) [103]. Quercetin inhibited the PI3-kinase activity and Cyanidin 3-*O*-glucoside also provided significant protection against cognitive dysfunctions that are induced by the administration of Aβ in animal models, mediated by the modulation of GSK-3b/tau. [104,105].

Overall, flavonoids may exert their potential neuroprotective actions by interacting with key proteins that are involved in AD. Better understanding the flavonoid−protein interactions in AD could be a promising strategy for developing novel neuroprotective therapies for the prevention and treatment of neurodegenerative diseases.

### 3.3. Flavonoids Interactions with Key Proteins and Lipoproteins in Atherosclerosis 

Atherosclerosis is another disease that flavonoids have been shown to attenuate. The first step in atherosclerosis is the accumulation of low-density lipoprotein (LDL), the main cholesterol carrier, in the arterial wall. High-density lipoproteins (HDL), on the other hand, is a major antiatherogenic factor in the blood, which maintains the whole-body level of cholesterol in a steady state. Over 80 proteins have been identified in the HDL proteome, with apolipoproteins A1 and A2 accounting for approximately 65% and 15% of the protein mass, respectively. The other proteins include a variety of enzymes, such as paraoxonase 1 (PON1). PON1 is responsible for many of HDL’s antiatherogenic properties. Correlations between PON1, HDL, and atherosclerosis, both in vivo and in vitro, have been well-established [106,107]. Aside from cholesterol efflux, HDL has other potent biological activities: antioxidative [108], anti-inflammatory [109], antiapoptotic [110], and vasodilatory [111]. These activities do not necessarily depend on HDL quantity, but they probably depend on its quality [112,113].

With respect to cardiovascular health, we have previously shown that the flavonoid glabridin, extracted from licorice root, acts as an excellent antioxidant and demonstrates additive antioxidant and antiatherogenic properties. Glabridin binds to recombinant PON1 (rePON1) and protects its Cys284 from oxidation by the atherosclerotic component linoleic acid hydroperoxide (LA-OOH). This specific capacity of glabridin is unique; the flavonoid catechin does not show any binding affinity to rePON1 [21]. The association between flavonoids structure and their effects on rePON1 activity was further explored. The interactions of 12 representative flavonoids from different chemical subclasses with rePON1 were characterized [42]. In addition, the potential of rePON1–flavonoid complexes to prevent oxidation of LDL, a key process in atherogenesis, was examined. Catechin, which does not bind to rePON1, accelerated LDL oxidation; in contrast, glabridin demonstrated a high binding affinity to rePON1 and enhanced its protective effect against LDL oxidation [42]. Moreover, we have consistently observed interactions of specific flavonoids with the HDL particle or its bound proteins, apolipoprotein A1 and PON1. We have shown that quercetin and punicalagin bind to the HDL particle and increase its anti-inflammatory properties [41], whereas, upon binding to the LDL particle or to its bound apolipiprotein B100, punicalagin induced LDL influx to macrophage J774A.1 cells, which may decrease the circulating LDL levels [114].

Overall, flavonoids, and polyphenols in general, have been found to inhibit symptoms of atherosclerosis and reduce its development via specific flavonoids interactions with cell and serum proteins and lipoproteins.

### 3.4. Flavonoids as Anticancer Agents via Interaction with DNA and Chromatin 

Flavonoids’ anticancer activities might be a result of the interaction of these natural compounds with biomolecules (DNA, RNA, and protein). We recognize that dietary flavonoids can bind the DNA specifically or stochastically and change its function [115]. Extensive in-vitro studies suggest that flavonoids effectively decrease cell proliferation, induce apoptosis, and lower the risk of metastasis [24]. The chemo-preventive effects of flavonoids, including luteolin, epigallocatechin gallate, quercetin, apigenin, and chrysin, were shown with a focus on protection against DNA damage that is caused by various carcinogenic factors. Those flavonoids selectively protect normal cells and induce cell-death mechanisms in cancer cells in human lungs and colorectal adenoma cells during chemotherapy or radiotherapy [24]. It was found that flavonoids, namely quercetin, myricetin, kaempferol, apigenin, and luteolin, which are lipid-soluble and weakly acidic, can freely diffuse across the cell membrane and specifically accumulate inside K562 leukaemic cells [116]. Therefore, it is implied that flavonoids are more likely to bind DNA or proteins in the cancer cell nucleus and to specifically interrupt cancer genome regulation. In addition, in-silico results have shown that quercetin, in particular, interacts well with G-quadruplex DNA, which is related to telomerase. Quercetin acts as a therapeutic anticancer agent via the regulation of telomerase activity [117]. By comparing computational and experimental binding profiles, a novel study confirmed that quercetin has the strongest binding affinity to DNA among the studied flavonoids. Furthermore, the study revealed that flavonoids can alter the conformation of DNA and inhibit DNA amplification, they show impressive induction of cell-cycle arrest, and they may promote apoptosis in HepG2, MCF-7, and A549 cancer cells [60].

In order to achieve the effective therapeutic doses used in preclinical studies, importance must be given to improved and targeted drug delivery techniques, so as to achieve maximum efficiency with minimal adverse side effects. Advances in nanotechnology-based drug delivery systems open up better opportunities for increasing solubility, improving bioavailability, and enhancing the targeting capabilities of flavonoids [118]. Nanoparticles based on liposomes, poly-ethylene glycol liposomes, nickel-based, lecithin-based, and nanoribbon are suitable molecular carriers for the delivery of flavonoid drugs to target tissues. It was reported that nanoparticles successfully used to deliver quercetin into solid tumors in in vitro and in vivo models of cancers of the central nervous system, lungs, colon, liver, and breasts [119].

Thus, numerous studies support the potential of flavonoids as natural health products in cancer chemoprevention. However, more studies are needed in order to configure their mechanism of action to improve our understanding of epigenetic processes that may provide a more rational basis for combining specific dietary compounds in a clinical setting [24].

## 4. Conclusions

Flavonoids are antioxidant molecules that are constantly consumed in our diets; they are found in our cells and serum. Therefore, it is interesting and highly important to determine their biological functions and mechanisms of action. Literature is mainly using classical antioxidant mechanisms to explain those molecules’ various cell and serum functions. However, it is more than reasonable that small, hydrophobic molecules at low concentrations will penetrate cytoplasm, organelle, and nucleus membranes and accumulate in specific cells and tissues to bind proteins via different types of interactions and affect signal transduction, gene expression, chromosomal alterations, epigenetic modifications, and so on. 

Further investigation at the proteome and genome levels is needed in order to characterize the mode of action by which flavonoids are involved in the reviewed OS related diseases. Mapping the direct interactions of flavonoids with proteins and chromatin genome-wide could provide new insights into the mechanisms by which flavonoids influence cellular functions, and pave the way to understanding, predicting, and controlling flavonoid responses in humans.

## Figures and Tables

**Table 1:** Methods used to characterize flavonoids-macromolecules interactions.

Macromolecule	Method	Type of Interaction	Reference
Flavonoids-Proteins interactions	UV-visible spectroscopy	Covalent complex formation or Protein conformational changes	[37]
	*Circular dichroism spectroscopy (CD)*	Proteins conformations and secondary structure changes	[38]
	*Fourier transform infrared spectroscopy (FTIR)*	Proteins conformations and secondary structure changes	[38]
	*isothermal titration calorimetry (ITC)*	Thermodynamic properties of the binding interaction between flavonoids and proteins	[39]
	*Taylor dispersion surface plasmon resonance (SPR)*	Binding affinities of non-covalent interactions	[40]
	*Tryptophan (Trp)-fluorescence quenching assay*	Determines interactions between flavonoids and proteins, quantitative analysis of binding affinities and thermodynamic parameters	[21,41,42]
	*docking calculations*	Computational models to predict the fit of the evaluated ligand within the protein,	[43]
Flavonoids-DNA interactions	electrochemical and SPR techniques, linear dichroism, absorption, fluorescence and nuclear magnetic resonance spectroscopies	Noncovalent interactions between flavonoids and DNA	[44,45,46]
	Electrospray ionization mass spectrometry (ESI-MS)	Binding of flavonoids with DNA duplexes	[47]
	Chemical affinity capture coupled with massively parallel DNA sequencing (Chem-seq)	Extracts and sequences DNA regions and captures chromatin regions bound to flavonoids.	[48,49,50]
